# Surgical Outcomes of Clival Chordoma Through Endoscopic Endonasal Approach: A Single-Center Experience

**DOI:** 10.3389/fendo.2022.800923

**Published:** 2022-04-06

**Authors:** Ge Chen, Mingchu Li, Wenlong Xu, Xu Wang, Ming Feng, Renzhi Wang, Xiaohai Liu

**Affiliations:** ^1^ Department of Neurosurgery, Xuanwu Hospital Capital Medical University, Beijing, China; ^2^ Chinese Pituitary Specialists Congress, Beijing, China; ^3^ Department of Neurosurgery, Peking Union Medical College Hospital, Chinese Academy of Medical Sciences and Peking Union Medical College, Beijing, China

**Keywords:** clival chordoma, endoscopic transnasal approach, surgical outcome, surgical complications, single center experience

## Abstract

**Objective:**

Clival chordoma is a locally aggressive tumor with low metastatic potential. In the past decade, endoscopic endonasal approach (EEA) for clival chordoma has had a higher resection rate and a lower morbidity rate than transcranial approaches. Here, we present our initial single-center experience after EEA of clival chordomas.

**Patients and methods:**

This study retrospectively analyzed 17 consecutive patients with clival chordoma who received EEA in our department between March 2015 and September 2021. The operation was performed by a single surgeon with EEA. The clinical and pathological characteristics were analyzed along with the surgical outcomes and complications.

**Results:**

A total of 17 consecutive patients with clival chordoma received EEA with a median follow-up of 29.2 months (range 1-79). Gross total resection (GTR) was performed in 7 cases (41%), subtotal resection (STR) in 7 case (41%) and partially resection (PR) in 3 cases (18%). Cerebrospinal fluid leakage occurred in 2 cases (12%) and meningitis developed in 3 patients (18%) which were all successfully treated with intravenous antibiotics without any complications. There were no perioperative deaths or new focal neurological deficits postoperatively. Four in 7 patients with STR have had radiotherapy while the other three chose to be monitored. Till the last follow-up, three patients in STR group who received radiotherapy (3 in 4) had no tumor regrowth, while one in STR group with radiotherapy (1 in 4) showed tumor progression. Two patients in STR group without radiotherapy (2 in 3) showed stable tumor while the left one (1 in 3) showed tumor progression. One patient in the PR group died of tumor progression 2 years postoperation and the other one showed tumor progression and died of lung cancer 1 year postoperation. In addition, 1 in 7 patients with GTR had tumor recurrence *in situ* after 10 months and developed surgical pathway seeding in the spinal canal in C1 after 16 months. No recurrence occurred in the other 6 cases with GTR during the follow-up.

**Conclusion:**

Although more cases are needed, our case series showed EEA is a safe and reliable method for clival chordoma with high resection rates and low morbidity rates. GTR without tumor residuum would improve the outcome.

## Introduction

Chordomas are rare and locally invasive bone tumors originating from remnants of the embryologic notochord with an incidence of approximately 0.1/100,000/year (1). The most common site of chordoma is the sacrococcygeal region (50%), followed by the skull base (35%), especially the clival region and vertebra (15%) (2). Although clival chordomas account for only 0.2% of all central nervous system tumors, they are characterized by local destruction, dural invasion, bone erosion, and cranial nerve palsy, and even metastasis, resulting in challenges for the surgical removal of this lesion. Moreover, local recurrence rates of clival chordoma are very high even after radical resection and adjuvant radiotherapy (3, 4), while chemotherapeutic agents are rare and are largely ineffective (5).

Most recently, endoscopic endonasal approach (EEA) was recommended as a first-line option with a higher resection rate and a lower surgical complication rate compared to the transcranial surgery for the treatment of clival chordomas, which is not yet widely accepted yet (6, 7). In this study, we aimed to report our single-center experience and surgical outcomes after EEA for the resection of clival chordomas, and showed that EEA provided a safe and reliable method for the resection of clival chordoma.

## Patients and Methods

### Patients

A total of 17 consecutive patients with clival chordoma who underwent EEA in Beijing Xuanwu Hospital between January 2015 and December 2021 were retrospectively analyzed. All the patients included had complete clinical, radiological, and biochemical data, over 18 years old, were treated with EEA and had at least half a year of follow-up. Histopathological and immunohistochemical examinations confirmed clival chordoma in all patients. The surgical procedures were performed by a single neurosurgeon (Ge Chen) with the surgical goal of total tumor resection. MRI was performed in all patients pre- and 1 week, 3 months, 6 months and every year post-operatively. The tumor size was measured as the maximum tumor diameter on MRI. The study was approved by the Research Ethics Committee of our hospital and written informed consent was obtained from all patients.

### Surgical Procedures

All EEAs were performed endoscopically using a Karl Storz endoscope through bi-nostrils, following the protocols described by the Pittsburg group (8). Hadad-Bassagasteguy (H-B) vascularized septal flaps with blood supply were performed in all cases (9). Septostomy and large sphenoidotomy were performed to provide access to the sellar area and clivus. Further surgical approaches depend on the location of the tumor and its upper and lateral extent. During the operation, we tried to detect the pseudocapsule of the tumor before the tumor resection. The adjacent dura mater was resected in all cases to achieve maximum surgical resection. All patients underwent multilayer closure with dura implantation, fat transplantation (when necessary), and a vascularized H-B flap. The internal carotid artery and basal artery were confirmed using Doppler in the patients with vascular invasion. Cerebrospinal fluid lumbar drainage was given postoperatively in all cases and was removed within 7 days after surgery. The MRI scans were evaluated by a neuroradiologist and another endoscopic surgeon to determine the extent of the tumor removal.

### Statistical Analysis

Statistical analysis was performed with SPSS 18.0 software (SPSS, Inc., Chicago, IL, USA). Descriptive statistics were performed to report data related to patients’ demographics and clinicopathological characteristics.

## Results

### Patient Characteristics

**Figure 1:**
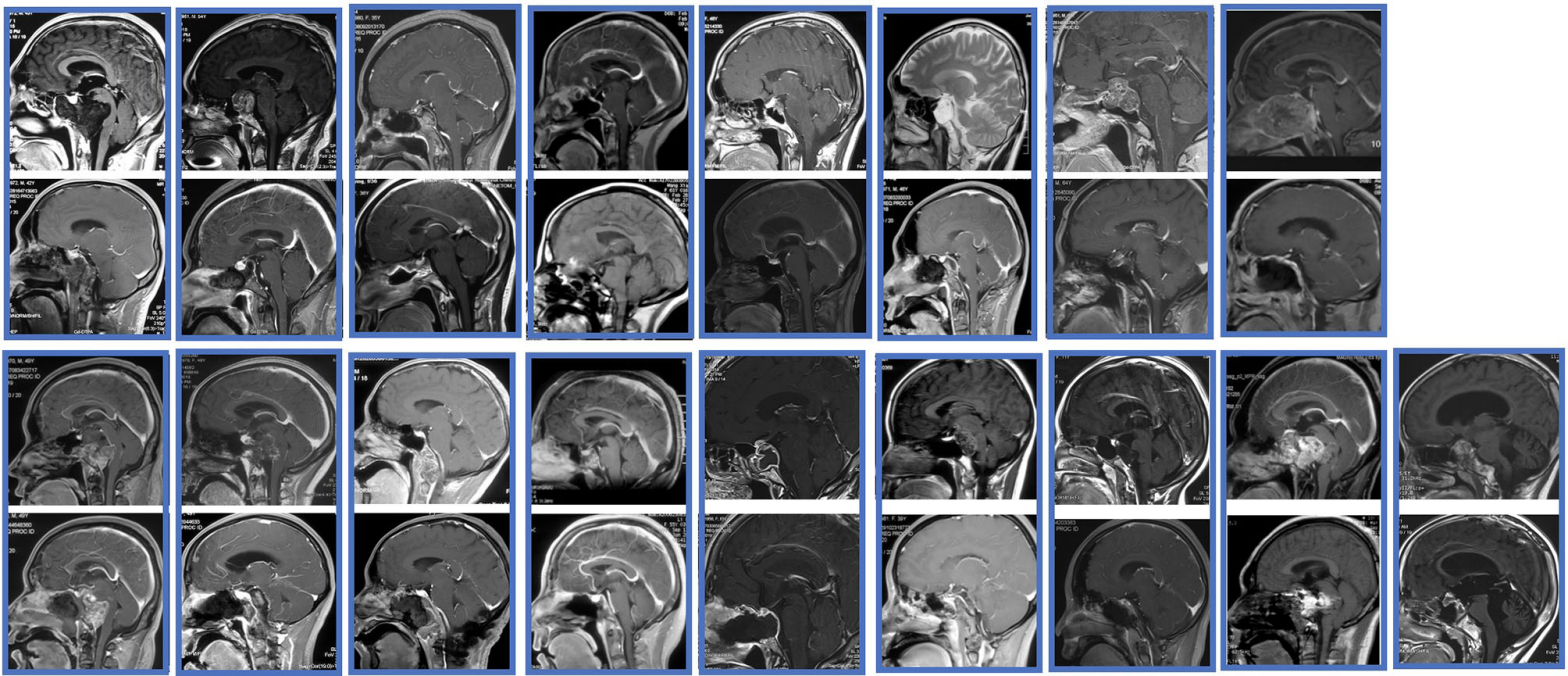
The pre- and post- MRIs of all the patients.

A total of 17 EEAs were performed in 17 patients. Three patients received one or two craniotomies while no one received radiotherapy before EEA and 2 cases with preoperative occipitocervical fusion surgery. The median age at diagnosis was 45 years and ranged from 10 to 67 years. The male to female ratio is 7:10. Nine patients out of 17 (53%) had headache, and eight patients were diagnosed with visual impairment or field defect (47%). Diplopia was the third most common symptom, which occurred in 6 patients (35%). Two patients exhibited dysphagia and dysphonia (12%) and hypophysis were seen in five patients at the time of diagnosis (29%). The average time from symptoms onset to diagnosis were 10.6 months (range 0.5–24 months). The maximum diameter and location of tumors are shown in **Table 1**. The maximum diameter of the 17 tumors arranged from 24.4 to 83.4 mm, with the mean diameter 45.9 mm. The mean tumor volume was 49.7 cm3 (range 3.1–220.5 cm3). Fourteen tumors (82%) were solid while only three were cystic. The tumor involved the clivus in 15 cases (88%), the sphenoid sinus in 6 cases (35%), the sella in 11 cases (65%), suprasella in 3 cases (18%) and in cavernous sinus 6 cases (35%). In one case, the tumor was located extended to the craniocervical junction. The pre- and post- MRIs of all the patients were shown in **Figure 1**.

**Table 1:** Clinical characteristics and surgical outcomes of the 17 patients with clival chordoma.

Case No.	Sex/Age	Symptoms	Tumor Location	Pituitary function	Tumor size(cm)	Tumor volume (cm^3^)	Surgical Outcome	Tumor residuum	Ki-67	Complications	Management of complication	Adjuvant radiotherapy	Follow up (Months)	Last imaging result
1	M/42	Headaches, vision loss of right eye, dysphagia and dysphonia	Sphenoid sinus, upper and middle clivus, right cavernous sinus, subdural	Normal	6.2	71.4	STR	right cavernous sinus, tumor attached to the basal artery	10	Intracranial Infection	Antibiotics	Gamma Knife	79	Recurrence
2	F/54	Vision loss and visual field defect	Sphenoid sinus, sella, upper clivus	Normal	3.9	23.8	GTR	—	5	—	—	—	69	No Recurrence
3	F/35	Headaches and, diplopia, CN 6^th^ nerve palsy	right cavernous sinus, upper and middle clivus, subdural	Normal	3.7	21.0	STR	right cavernous sinus	5	—	—	Proton knife	64	Stable
4	F/60	Headaches, vision loss of right eye and two craniotomies	Anterior cranial fossa, right orbital cavity	ND	5.2	59.8	PR	Anterior cranial fossa, right orbital cavity	15	—	—	—	12	Tumor progression and died of lung cancer
5	F/48	Headaches and vision loss of both eyes	Sella and suprasella, subdural	Normal	2.4	3.8	GTR	—	3	—	—	—	50	No Recurrence
6	M/49	Headaches	Sella, right cavernous sinus and upper clivus, subdural	ND	3.4	12.4	GTR	—	2	Intracranial Infection	—	—	44	No Recurrence
7	M/67	Headaches, vision loss of right eye and one craniotomy	Sphenoid sinus, sella, upper and middle clivus	Low	3.0	11.1	GTR	—	3	Intracranial Infection	—	—	25	No Recurrence
8	M/62	Vision loss of both eyes	Sphenoid sinus, sella, upper and middle clivus	Low	8.3	220.5	STR	—	3	—	—	—	25	Stable
9	M/49	Diplopia, CN 3^rd^ and 6^th^ nerves palsy	Sella, left cavernous sinus and upper, middle and lower clivus, subdural	Low	6.6	79.0	PR	left cavernous sinus	20	—	—	—	24	Died of recurrence
10	F/49	gait instability	Sella, upper and middle clivus, subdural	Low	6.7	115.4	GTR	—	5	—	—	Gamma Knife	22	Tumor recurrence and Intradural spinal seeding
11	M/38	Headaches, stiff neck, dysphagia and dysphonia	upper, middle and lower clivus, craniocervical junction, subdural	ND	5.2	59.9	STR	—	20	—	—	Gamma Knife	17	Stable
12	F/55	Diplopia	Sella, right cavernous sinus and upper and middle clivus, subdural	ND	3.2	12.5	STR	right cavernous sinus	10	—	—	Proton knife	16	Stable
13	F/63	Headaches and vision loss of both eyes	Sphenoid sinus, sella and suprasella, upper and middle clivus, intradural	Normal	4.4	24.4	STR	suprasella	5	—	—	—	28	Recurrence
14	F/39	Diplopia, left CN 6^th^ nerve palsy	upper and middle clivus, intradural	Normal	4.1	20.7	GTR	—	5	—	—	—	12	No Recurrence
15	F/11	Diplopia, left CN 6^th^ nerve palsy	upper and middle clivus, intradural	Low	3.2	11.2	GTR	—	3	—	—	—	6	No Recurrence
16	M/33	Headaches and vision loss	Sphenoid sinus, sella and suprasella, upper and middle clivus, subdural	Normal	7.5	71.4	PR	subdural	5	—	—	—	1	Stable
17	F/10	Diplopia, CN 6^th^ nerve palsy and two craniotomies	Sella, left cavernous sinus, upper and middle clivus, subdural	Normal	4.8	26.9	STR	subdural	5	—	—	—	3	Stable

ND, Not done.

### Surgical Outcome

Gross total resection was performed in 7 cases (41%), STR in 7 case (41%) and PR in 3 case (18%) (**Figure 1**). The mean maximum diameters of the GTR, STR, and PR groups were 38.1, 51.1, and 64.3 mm, respectively. The value in the GTR group was significantly smaller than in the other 2 groups by multiple comparisons (P < 0.001, P < 0.001, respectively). The residual tumor was either in the cavernous sinus or in the subdural space, which could be adhered tightly to the dura and to the important vessels, such as the basal artery. Another reason for the tumor residual was due to the texture of the tumor. Headache and visual impairment or field defect were improved in most of the patients while five patients with the symptom of diplopia (5 in 6) resolved within 6 months after surgery. All the two patients with dysphagia and dysphonia were recovered within one year postoperation. The Ki-67 index ranged from 3-20%, with a mean value of 7.6%, showing its active proliferation capacity. Interestingly, the residual tumor in one of the three patients in the PR group showed rapid tumor growth in the follow up and died 12 months after surgery with a Ki-67 index of 20%.

### Surgical Complications and Follow-Up

As the tumor invaded the dura in 13 patients, the repair using the fascia of thigh muscle and H-B flap were performed in these patients. A second lumbar drainage after operation was used to manage the cerebrospinal fluid leakage. One patient developed cerebrospinal fluid leakage 2 weeks after the operation and required a secondary endoscopic repair. Postoperative bacterial meningitis was found in 3 patients who had no significant cerebrospinal fluid leakage. All three patients were successfully treated with intravenous antibiotics without any complications. One patient who received GTR developed deterioration of pituitary function after the EEA and received adrenocorticoid and thyroid hormone replacement therapy, while one in four patient who had pituitary function failure had normal function in the last follow-up. Two patients with GTR developed temporary diabetes after the EEA and were successfully treated with desmopressin. None of these patients developed persistent diabetes or new-onset neurological dysfunction. All the 7 patients with STR were considered for adjuvant radiotherapy, while only 4 of them have had radiotherapy and the other three chose to be monitored, including one female patient aged 10 years who were not suitable for radiotherapy. Till the last follow-up, three patients in STR group who received radiotherapy (3 in 4) had no tumor regrowth, while one in STR group with radiotherapy (1 in 4) showed tumor progression. Two patients in STR group without radiotherapy (2 in 3) showed stable tumor while the left one (1 in 3) showed tumor progression. One patient in the PR group died of tumor progression 2 years postoperation and the other one showed tumor progression and died of lung cancer 1 year postoperation. In addition, 1 in 7 patients with GTR had tumor recurrence *in situ* after 10 months and developed surgical pathway seeding in the spinal canal in C1 after 16 months. No recurrence occurred in the other 6 cases with GTR during the follow-up.

## Discussion

Chordomas were first described by Virchow in 1857 (10), while the term chordoma was first proposed by Ribbert in the 1890s, representing the microscopic characteristics of the tumor derived from undifferentiated notochordal remnants (11). According to the Surveillance, Epidemiology and End Results (SEER) database, the incidence of chordomas is 0.08 per 100,000, which occurs mainly in males, and occurs between 50 and 60 years of age, with a median survival of 6.29 years (12). Although chordomas represent only 0.2% of all central nervous system tumors, the most common site of origin is the clivus for intracranial chordomas. Because they are characterized by local destruction, dural invasion, bone erosion, and cranial nerve palsy, and even metastasis, clival chordomas are difficult to manage and easily recur, resulting in poor prognosis (13).

Chordomas may be located on the upper clivus, along the caudal edge of the clivus, the sellar area, sphenoid sinus, nasopharynx, maxilla, or even the intradural area (14). The clinical manifestations are varied and are associated with the location and involvement of adjacent structures of the clival chordoma. Patients usually present with headache, diplopia and vision loss. In rare oropharyngeal manifestations, dysphagia and speaking difficulties are present, which is common in clival chordoma with parapharyngeal and retropharyngeal extension (15). Although a definitive diagnosis of clival chordoma requires histopathological results with the characteristic material-like appearance of cells, typical clinical signs should be evaluated for the early diagnosis and treatment (16).

Although the lateral transcranial approach provides better vascular control and a better view of the brainstem-tumor interface (17), some have argued that EEA may not only provide direct surgical access but also provide a better visualization of surrounding structures, which is safer and minimally invasive (18). In the past decade, the use of EEA has been recommended more with increasing indications and better results (12). Here, we present our initial single-center experience and short-term outcomes after endoscopic endonasal approach resection of clival chordomas.

As surgery is the first-line treatment for clival chordoma, there are many surgical approaches for chordoma resection. Traditional transcranial approaches often lead to more brain tissue retraction with increased cerebral edema, hematoma and more damage to surrounding structures such as the basilar artery and optic nerve (19). As clival chordoma is located in the middle line of the skull base, EEA for clival chordoma resection can easily provide the surgical pathway and excellent exposure of the tumor and adjacent structures (especially the anterior dura and basilar arteries) (20, 21). The use of angled endoscopy has an advantage in showing hidden areas that cannot be seen with the transcranial approach. Zhang et al. proposed a surgical strategy for EEA according to the tumor growth directions, contributing to increasing the GTR rate (22). Therefore, EEA for clival chordoma resection has less morbidity compared to the transcranial approach (23). In our study, EEA resulted in a high GTR rate and a low surgical complication rate. Although intraoperative cerebrospinal fluid is very common, it is still inevitable during endoscopic surgery as cerebrospinal fluid repair could be performed after tumor resection with improved reconstructive techniques.

When the neurovascular structures of the surrounding area are locally invaded by the clival chordoma, the surgical principle is to minimize neurological dysfunction, even at the expense of having a postoperative residual tumor (12). After aggressive resection, radiotherapy could be used, with which residual tumors, especially small ones, can be effectively treated by radiotherapy. In 19 patients who underwent surgery and postoperative stereotactic radiotherapy, high-dose radiotherapy effectively controlled the small residual tumor volume (24). In a previous report, eleven patients underwent chordoma resection, of which 7 had subtotal or partial resections (24). Transient neurological deterioration (cranial nerve defects) occurred in seven patients, all of whom returned to neurological baseline. Following these considerations, 2 patients developed diplopia postoperatively but both returned to normal within 6 months in our case series, providing the evidence that the surgical strategies should not be overly aggressive, but should consider the option of radiotherapy and residual tumor observation.

Local recurrence is the main form of treatment failure (25). Radiotherapy can be used to treat recurrent clival chordoma patients who are not suitable for surgery (26). En bloc excision plus high dose radiation (27) or en bloc resection with proton beam radiation (28) were both the best evidences for improving survival in these patients. In our case series, one patient showed a recurrence *in situ* after EEA and gamma knife treatment was performed. After a follow-up of 16 months, a new lesion was seen in the spinal canal at C1 to C2, where an intradural spinal seeding chordoma was highly suspected. After a craniotomy, the lesion in the spinal canal was totally removed and the pathology confirmed a chordoma with increasing proliferative potential. And the patient was still in intensive follow-up.

Although the pathogenesis of chordoma is still unclear, loss of heterozygosity (LOH) of 1p36 is very common among sporadic chordomas and is related to tumorigenesis (29). As the molecular pathogenesis of clival chordoma is still unknown, no chemotherapy or targeted therapy has been developed until now. There is no specific genetic biomarker involved in predicting the recurrence and metastatic potential of chordomas. Although long-term prospective studies should be carried out to evaluate the role of endoscopic endonasal surgery in clival chordoma, endoscopic endonasal resection of clival chordoma is minimally invasive and reliable, which is correlated with a high GRT rate and a low morbidity.

## Conclusion

Although more cases are needed, our case series showed EEA is a safe and reliable method for clival chordoma with high resection rates and low morbidity rates. GTR without tumor residuum would improve the outcome.

## Data Availability Statement

The raw data supporting the conclusions of this article will be made available by the authors, without undue reservation.

## Ethics Statement

The studies involving human participants were reviewed and approved by Ethics Committee of Xuanwu Hospital Capital Medical University, Written informed consent to participate in this study was provided by the participants’ legal guardian/next of kin.

Written informed consent was obtained from the individual(s), and minor(s)’ legal guardian/next of kin, for the publication of any potentially identifiable images or data included in this article

## Author Contributions

Design, provision of patients, collection and assembly of data, data analysis and interpretation, manuscript writing. All authors contributed to the article and approved the submitted version.

## Funding

The financial support for this study was provided by Beijing Hospitals Authority Youth Program (Code: QMS20210802 to XL).

## Conflict of Interest

The authors declare that the research was conducted in the absence of any commercial or financial relationships that could be construed as a potential conflict of interest.

## Publisher’s Note

All claims expressed in this article are solely those of the authors and do not necessarily represent those of their affiliated organizations, or those of the publisher, the editors and the reviewers. Any product that may be evaluated in this article, or claim that may be made by its manufacturer, is not guaranteed or endorsed by the publisher.

## Abbreviations

EEA, endoscopic endonasal approach; GTR, Gross total resection; PR, partially resection; STR, subtotal resection.
